# Early High-Fat Diet Exposure Causes Dysregulation of the Orexin and Dopamine Neuronal Populations in Nonhuman Primates

**DOI:** 10.3389/fendo.2018.00508

**Published:** 2018-09-10

**Authors:** Cadence True, Anam Arik, Sarah Lindsley, Melissa Kirigiti, Elinor Sullivan, Paul Kievit

**Affiliations:** ^1^Cardiometabolic Health Division, Oregon National Primate Research Center, Beaverton, OR, United States; ^2^Division of Neuroscience, Oregon National Primate Research Center, Beaverton, OR, United States; ^3^Department of Human Physiology, University of Oregon, Eugene, OR, United States

**Keywords:** obesity, orexin, dopamine, appetite, high-fat diet, nonhuman primate

## Abstract

Maternal obesity and consumption of a high-fat diet (HFD) during pregnancy has a negative impact on offspring, including an increased risk for the development of obesity in adolescence. The mechanism for this transferred metabolic risk is unclear, but many studies have focused on the brain due to its important role in appetite and body-weight regulation. Two main pathways regulate appetite in the brain; homeostatic regulation that occurs predominantly in hypothalamic circuits and hedonic regulation of feeding that occurs via dopaminergic pathways. The current proposal examined the impact of early HFD exposure on the dopaminergic control of hedonic feeding pathways in a translational nonhuman primate model. Japanese macaque offspring from mothers consuming a control (CTR) or HFD were weaned onto control or HFD at an average 8 months of age yielding four groups: maternal and post-weaning control diet (mCTRpCTR), maternal control diet and post-weaning HFD (mCTRpHFD), maternal HFD and post-weaning control diet (mHFDpCTR) and maternal and post-weaning HFD (mHFDpHFD). Brains from 13-month-old offspring were evaluated for expression of neuropeptides that regulate dopaminergic pathways including orexin, melanin-concentrating hormone (MCH) in the lateral hypothalamus (LH), and tyrosine hydroxylase expression in the ventral tegmental area (VTA). Orexin cell numbers in the LH were significantly increased in animals exposed to a post-weaning HFD, while no difference was observed for orexin mRNA content or MCH cell numbers. Orexin fiber projections to the rostral VTA were significantly reduced in mCTRpHFD, mHFDpCTR, and mHFDpHFD groups, but these differences were not significant in the caudal VTA. There was no difference in the percentage of dopamine neurons receiving close appositions from orexin fibers in either the rostral or caudal VTA, nor was there any difference between groups in the number of orexin contacts per TH cell. In conclusion, the current study finds that prolonged early exposure to HFD during the *in utero* and postnatal period causes alterations at several levels in the dopaminergic circuits regulating reward.

## Introduction

Maternal obesity and diabetes are associated with an increased risk of childhood obesity in offspring (1–3). Given that an estimated 41% of women in the United States are obese (4), this represents a significant health risk for future generations (5, 6). The mechanism for this conferred risk remains unclear. One hypothesis is that maternal obesity programs the offspring during critical periods of *in utero* development, which predisposes them toward obesity in adulthood. However, given the myriad of organs that participate in regulating body weight, it remains unclear where this programming may occur. Our laboratory has developed and characterized a model of early high-fat diet (HFD) exposure in nonhuman primates to investigate where and how this disposition for obesity might arise (7–9).

The brain plays a critical role in the regulation of body weight, particularly in modulating appetite. Regulation of food intake is controlled by two interacting pathways in the brain: those controlling metabolic homeostasis and those encoding food reward (10–12). Alterations in both pathways are associated with increased food intake and obesity (13–16). Previous work has illustrated that maternal HFD consumption and obesity elicits changes in offspring expression of neuropeptides in the hypothalamus, which play a critical role in regulating appetite homeostasis (7, 9, 17, 18). Importantly, these studies find maternal obesity and/or HFD consumption decreases offspring expression of hypothalamic orexigenic factors like neuropeptide Y and agouti-related peptide that stimulate food intake and decreases in hypothalamic anorexigenic factors like proopiomelanocortin which decrease food intake. In addition to alterations in these homeostatic pathways, previous work from our laboratory has reported that maternal HFD consumption in a nonhuman primate model results in increased hedonic feeding during a food preference test, which was associated with decreased dopaminergic fibers and receptors in the prefrontal cortex (19). Work in rodents has hypothesized that reward signaling is encoded in part by the neuropeptides of the lateral hypothalamus such as orexin, and to a lesser extent melanin concentrating hormone (MCH), regulating dopamine neurons in the ventral tegmental area (VTA) (20–22). VTA projections to the prefrontal cortex are hypothesized to encode higher reward processing. The current study utilized a nonhuman primate model of maternal and postnatal HFD exposure to determine whether changes in the lateral hypothalamus and VTA neuronal populations are causative for previously observed increases in hedonic feeding (19).

## Methods

### Animals

All animal procedures were approved by the Oregon National Primate Research Center (ONPRC) Institutional Animal Care and Use Committee. Adult female Japanese macaques were assigned to the study, with an average age of 7.9 ± 0.35 years ranging between the ages of 3.6–10.7 years. The average weight in the CTR fed group at the time of conception was 8.6 ± 0.4 kg with a range of 6.1–12 kg. The average weight in the HFD fed group at the time of conception was 11.8 ± 0.7 kg with a range of 7.5–17.1 kg. Dams were either maintained on a control diet (CTR, 15% calories from fat; Purina Mills Monkey Diet no. 5052) or placed on a HFD (37% calories from fat; Purina Mills TAD Primate Diet no. 5LOP) provided *ad libitum* for 1–5 years prior to offspring delivery and throughout lactation. Detailed dietary information has previously been described (7, 8, 23). Dual energy X-ray absorptiometry (DEXA) body composition analysis and intravenous glucose tolerance tests (ivGTT) were performed as described previously (8) in the early fall prior to pregnancy and are summarized in Supplemental Table 1, along with weight and age near the estimated time of conception. Obesity and insulin resistance did not necessarily follow dietary groups and for the current analysis, dams were solely separated by the diet they consumed. Female and male offspring were born naturally and remained on dam's diets until weaning. Average age of weaning was 259 ± 5.2 days, corresponding to ~ 8 months of age. Before weaning, animals were housed in either small group (4–12 individuals, male:female ratio of 1–3:10) or large groups (150–200 individuals) in indoor/outdoor enclosures. At weaning, all offspring were group housed in enriched indoor/outdoor environments with 6–10 similarly aged juveniles and 1–2 unrelated adult females per group. Offspring from both maternal diet groups were assigned to a postnatal diet of either CTR or HFD yielding four offspring groups: CTR/CTR *n* = 8 (5 females, 3 males), CTR/HFD *n* = 9 (4 females, 5 males), HFD/CTR *n* = 8 (4 females, 4 males), HFD/HFD *n* = 8 (4 females, 4 males). Offspring underwent ivGTTs at ~ 12–14 months of age and this data along with weight at ivGTT is summarized in Supplemental Table 1.

### Tissue collection and processing

Animals were necropsied at ~13–14 months of age and brain tissue was collected as previously described (19, 24, 25). Offspring were deeply anesthetized with a surgical dose of sodium pentobarbital (30 mg/kg i.v.) and then exsanguinated. Perfusion of the brain occurs via the carotid artery by flushing with 0.9% heparinized saline (0.5–1 L) followed by 4% paraformaldehyde (PF, ~ 1–2 L) buffered with sodium phosphate (NaPO4, pH 7.4) until fixed. The brain is then partitioned into specific areas and post-fixed followed by cryoprotection in glycerol and flash freezing in −50°C 2-methylbutane. Brains were stored in −80°C until sectioning (25-μm-thick; 1:24 series) using a sliding microtome. Sections were stored in cryoprotectant, and subsequent immunohistochemistry utilized every 12th section for analysis.

### Immunohistochemistry

All immunohistochemistry, imaging, and analysis was done blinded to animal groups. Coronal tissue sections were washed in 0.05 M potassium phosphate-buffered saline (KPBS) and incubated in blocking buffer (2% normal donkey serum + 0.4% Triton X-100 + KPBS) for 30 min at room temperature. Tissue sections were then incubated in a cocktail of primary antibodies for 24 h at room temperature. A mouse anti-tyrosine hydroxylase (TH; Millipore MAB218) was used at 1:1,000 to label dopamine neurons and has been previously validated in primate tissue (19, 26). A goat anti-orexin antibody (Santa Cruz, sc-8070) was used at 1:1,000 and has previously been validated (27). A rabbit anti-melanin concentrating hormone (MCH; Phoenix Pharmaceuticals, H-070-49) was used at 1:3K and has previously been validated (27). After incubation, tissues were washed in KPBS, and incubated for 1 h in the following Alexa fluorophore (1:1,000): TH – Donkey anti-mouse 647, Orexin – Donkey anti-goat 568, MCH – donkey anti-rabbit 488. Tissue was mounted on gelatin-coated slides and coverslipped with buffered glycerol.

### Imaging and analysis

#### Orexin, MCH and TH cell counts

For cell count analysis imaging was performed on an Olympus Slidescanner BX61VS using a 20x objective. Sequential imaging of fluorophores was performed to avoid bleed-through. For the lateral hypothalamus/posterior hypothalamic area, three sections per animal were imaged for both MCH- and orexin-immunoreactivity. Cells were counted for one side of the brain only. For the VTA, six sections were analyzed per animal for tyrosine hydroxylase (TH)-immunoreactivity. All TH cells visible along the midline were counted. MCH cells were easily identifiable; therefore, an automated macro was created in ImageJ to count MCH neurons. Briefly, a common threshold was set for all images and a mask was created. The “Fill Holes” function was utilized followed by Analyze Particles with 650-infinity pixel setting. Orexin and TH-cell bodies were intermingled with dense fiber networks and were counted manually using a common threshold for each antibody across all sections and confirmed with DAPI nuclear labeling.

#### Orexin fiber density

For fiber density analysis, 6 VTA sections per animal were imaged along the midline on a Leica SP5 AOBS confocal microscope (Leica Microsystems, Buffalo Grove, IL) using a 10X objective with 1 μM z-plane stacks. Fluorophores were again imaged sequentially to avoid bleed-through. A common threshold for all images was set for orexin immunoreactivity. Tyrosine hydroxylase-immunoreactivity was used to place the region of interest for analysis in the most dopamine rich VTA region for each section analyzed. Percent area of the region of interest that contained pixels with staining above threshold was calculated using ImageJ for ten 1 μM stacks with the highest intensity, then normalized for the tissue area analyzed.

#### Orexin close apposition to TH cells

For close apposition analysis, sections were imaged again on the Leica SP5 AOBS confocal microscope using a 40x objective (and zoom factor of 2) with 1 μM z-plane stacks. Two VTA sections were analyzed per animal, corresponding to the rostral most section which contained the largest disparity between groups in orexin fiber density (Section 1), and a more caudal section containing the largest disparity of VTA TH-IR neurons between the groups (Section 5). For each section, analysis was performed immediately left and right of midline per section, corresponding to the most TH cell dense region. The number of TH cells in each photomicrograph was counted manually and labeled. Each cell was then examined for close appositions by orexin-immunoreactive fibers using ImageJ. Images were scanned through individual 1 μM z-planes to ensure close apposition occurred on the same z-plane. A close apposition was defined as an abutment of an orexin-immunoreactive fiber that was present on all orthogonal views, and resulted in either an overlapping of pixels or an absence of negative (black) pixels between the labeling. The percent of TH-IR cells receiving close appositions as well as the average number of appositions per contacted cell were calculated independently for Section 1 and Section 5.

### *In situ* hybridization

*In situ* hybridization for orexin was performed on the same set of animals used for orexin IHC in the lateral hypothalamus and has been described previously (7, 28). Briefly, a 1:12 series of arcuate hypothalamic tissue was mounted onto slides in RNase free conditions for each animal. Brain sections were fixed in 4% paraformaldehyde (pH 7.4), treated with proteinase K at 37°C to increase penetration, and then with 0.25% acetic anhydride in 0.1 M triethanolamine (pH 8.0). Sections were then rinsed in 2X SSC, dehydrated through graded series of alcohols, delipidated in chloroform, rehydrated through a second series of alcohols and air-dried. The sections were exposed to a human orexin probe labeled with ^33^P−UTP overnight in a humidified chamber at 55°C. The probe was generated from a pBluescript II plasmid with an insert corresponding to base pairs 378–774 of the macaque orexin mRNA sequence (NM_001194432.1) and transcribed in the antisense direction (plasmid manufactured by Genscript). After incubation, the slides were washed in 4X SSC, in RNase A at 37°C, and in 0.1X SSC at 60°C. Slides were then dehydrated through a graded series of alcohols and dried. For visualization of the probe, labeled sections were exposed to film (Biomax MR, Kodak) overnight and imaged using a CoolSNAP charge-coupled camera (Photometrics) and analyzed using ImageJ. *In situ* hybridization mRNA labeling matched the distribution of orexin cells by immunohistochemistry as well as previously published description of orexin distribution in primates (29). Three lateral hypothalamus sections were analyzed unilaterally per animal. A common threshold was set for all images and a defined region of interest that incorporated one side of the lateral hypothalamus was utilized. The percent area of pixels over the threshold was quantified within the region of interest for three sections per animal and then averaged for each animal.

### Statistics

Analysis between the four groups was performed by two-way ANOVA for maternal and post-weaning diet factors and *post-hoc* Tukey comparison between the four groups in Prism GraphPad. Analysis of TH-IR cells and orexin fiber density across sections of the VTA was analyzed by repeated measures ANOVA for Tukey *post-hoc* comparison between the four groups for each section of the VTA. All data was checked for normality, with D'Agostino and Pearson normality test *p* > 0.05. MCH cell count was log transformed to pass normality testing; however, no difference in 2-way ANOVA analysis was observed with either log transformed or raw data, so raw data is presented. All data was checked for outliers, using Prism GraphPad outlier detector (setting Q = 2) and identified outliers were removed. All measurements were evaluated for the influence of offspring sex and maternal age and weight near conception in SPSS; however, these factors did not have significant main effects and were left out of final data analysis. All data are presented means ± standard error of the mean.

## Results

### Lateral hypothalamus orexin and MCH protein and mRNA content

**Figure 1:**
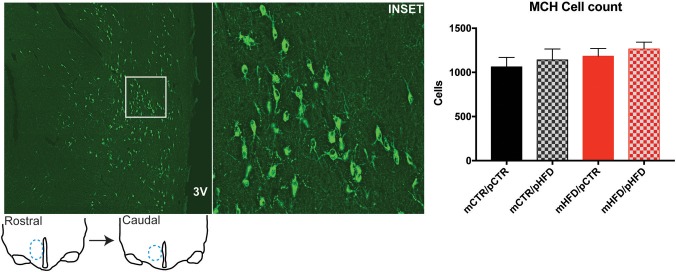
Quantification of melanin-concentrating hormone (MCH) cell bodies in the posterior hypothalamic area and lateral hypothalamus by immunohistochemistry. The number of MCH-IR (green) cell bodies was counted unilaterally across 3 sections/animal, rostral and caudal extent of sections analyzed are depicted in line drawings, with lateral hypothalamus region indicated in blue. Left photomicrograph is roughly 2,500 × 2,500 μm with the inset region shown in white, which is roughly 500 × 500 μm. Analyzed sections correspond roughly to Figure 59 from Paxionos Rhesus Atlas. 3V-third ventricle. *N* = 6–8 animals/group.

**Figure 2:**
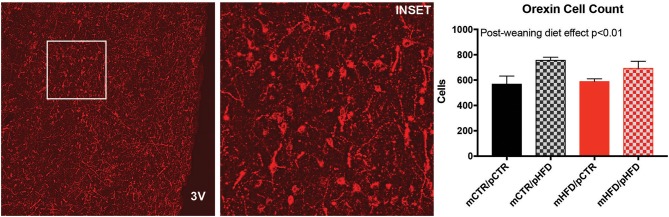
Quantification of orexin cell bodies in the posterior hypothalamic area and lateral hypothalamus by immunohistochemistry. The number of orexin-IR (red) cell bodies was counted unilaterally across 3 sections/animal corresponding to the same brain regions indicated in Figure 1. Left photomicrograph is roughly 1,800 × 1,800 μm with the inset region shown in white, which is roughly 500 × 500 μm. 3V- third ventricle. *N* = 6–8 animals/group. Statistically significant effects by two-way ANOVA are denoted as text within the graph.

**Figure 3:**
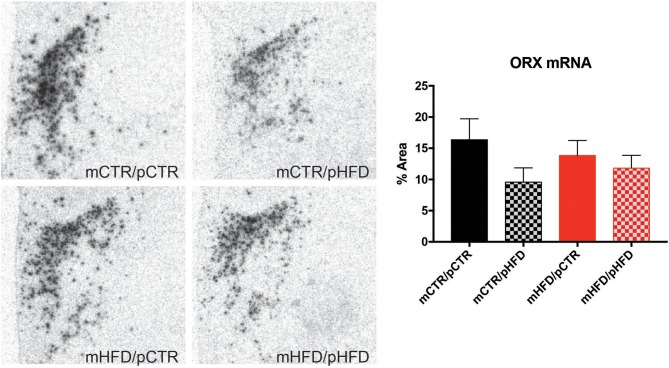
Quantification of orexin mRNA content in the posterior hypothalamic area and lateral hypothalamus by *in situ* hybridization. The percent area of pixels elevated over threshold was quantified unilaterally across 3 sections/animal corresponding to the same brain regions indicated in Figure 1. *N* = 6–8 animals/group.

Previous metabolic profiling of this animal model, which includes many animals in the current study, found that offspring from HFD-fed mothers weigh less initially but demonstrate rapid catch-up growth, and those that were weaned onto a HFD weighed more than control animals at 13 months of age (9). The specific metabolic parameters of the dams and offspring cohort utilized in the current study are provided in Supplemental Table 1. The current study sought to determine how these metabolic changes may be regulated by reward circuitry in the brain. MCH and orexin cell were identified in the region spanning the posterior hypothalamic area (PH) and lateral hypothalamus (LH) and counts were investigated using immunohistochemistry. There were no differences in the number of MCH cells in the PH/LH between the four groups (Figure 1). Post-weaning HFD consumption increased orexin-immunoreactive (-IR) cell number in the PH/LH [post-weaning diet, *F*_(1,26)_ = 9.12, *p* < 0.01], but there were no *post-hoc* differences between the four individual groups (Figure 2). Orexin mRNA content was also examined in the PH/LH by *in situ* hybridization. Surprisingly, there was no difference between the four groups for orexin mRNA content, indicating a discordance between protein content (quantified by immunohistochemistry counts of cell bodies) and overall mRNA content (Figure 3).

### Orexin and MCH projections to the VTA

**Figure 4:**
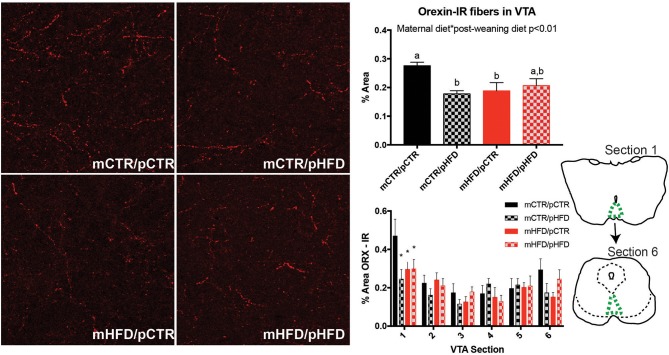
Quantification of orexin fibers in the VTA by immunohistochemistry. The percent area of a fixed region of interest with an intensity over a set threshold was quantified for orexin-IR in the VTA (top right). Fiber quantification was also broken down by VTA section (1–6; bottom right). Line drawings depict rostral (Section 1) and caudal (Section 6) extent of sections analyzed with VTA region indicated in green. Photomicrographs are approximately 380 × 380 μm. *N* = 7–8 animals/group. Different letters denote significant difference (*p* < 0.05). *denotes statistically significant difference between mCTR/pCTR group and the other three groups (*P* < 0.05). Statistically significant effects by two-way ANOVA are denoted as text within the graph.

Both MCH and orexin fiber projections to the VTA were examined. MCH-IR fibers were present in the brainstem, but largely absent from the VTA region where dopamine neurons are found (data not shown). Orexin-IR fibers were present in the VTA near dopamine neurons, analysis of average fiber density across the 6 sections analyzed revealed an interaction between maternal diet and post-weaning diet [*F*_(1,26)_ = 8.10, *p* < 0.01; Figure 4]. Both mCTRpHFD and mHFDpCTR groups had a significant decrease in orexin-IR fiber density in the VTA compared to the control group (*p*'s < 0.05). Analysis between individual VTA regions (Sections 1–6) revealed this reduction in orexin fiber density was predominantly within the rostral VTA (Section 1), where all three treatment groups showed decreased orexin fiber density compared to the mCTRpCTR group (*p*'s < 0.05).

### Dopamine content and orexin fiber contacts in the VTA

**Figure 5:**
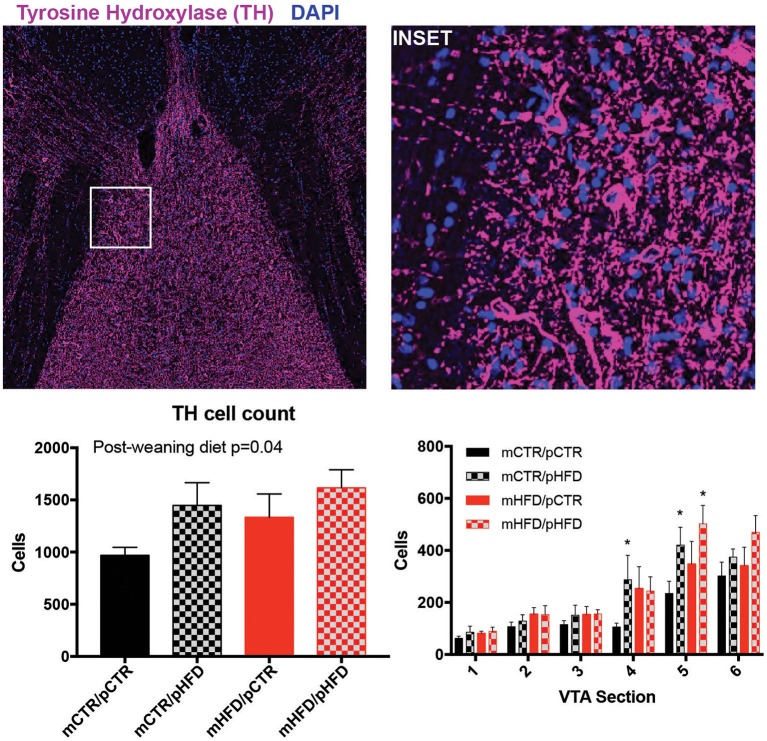
Quantification of tyrosine hydroxylase (TH) cells in the VTA by immunohistochemistry. TH-IR (magenta) cells were counted manually, confirmed by DAPI labeling (blue), across 6 VTA sections/animal. Cell count was also broken down by VTA section, with section number corresponding to the same depiction in Figure 4. Left photomicrograph is roughly 1,500 × 1,500 μm with the inset region shown in white, which is roughly 250 × 250 μm. 3V- third ventricle. *N* = 7–8/group. *denotes statistically significant difference from mCTR/pCTR (*p* < 0.05).

**Figure 6:**
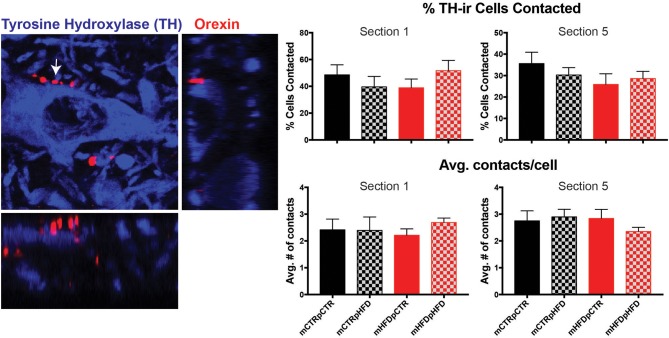
Quantification of orexin-IR fiber close apposition to tyrosine hydroxylase (TH) neurons in the VTA. Both the percent of TH-IR (blue) cells receiving close appositions from orexin-IR fibers (red) and the number of close appositions per contacted cell were analyzed in Section 1 and Section 5. Arrow indicates close apposition depicted in orthogonal views, shown to the right and below. Photomicrograph is approximately 50 × 50 μm. *N* = 7–8 animals/group.

The total number of TH-IR cells in the VTA was quantified for each group. Interestingly, the total number of TH-IR cells in the VTA was increased with post-weaning HFD exposure [post-weaning diet, *F*_(1,27)_ = 4.45, *p* = 0.04; Figure 5]. This increase appeared to be specific to the caudal portion of the VTA (Section 5), with dopamine cell numbers similar between groups in the rostral VTA (VTA section number corresponds to the same anatomical locations specified in Figure 4). To examine whether decreased orexin content in the VTA was specifically linked to decreased input to dopamine neurons, close appositions between orexin-IR fibers and TH-IR neurons were examined. Contact analysis was performed in Section 1 and Section 5, corresponding to the rostral and caudal VTA respectively, since this is where differences in orexin density and TH-IR cell numbers were observed. The percent of TH-IR neurons receiving orexin fiber close appositions was not different between the four treatment groups at either the rostral or caudal VTA. (Figure 6). Most TH-IR cells with close appositions had between 2 and 3 potential contacts/cell, which also did not differ across the four groups.

## Discussion

The current study finds that post-weaning consumption of a HFD alters the reward neurocircuitry of the brain in nonhuman primates. Specifically, increases in orexin-IR cells in the LH and TH-IR cells in the VTA were observed; however, orexin mRNA content was unchanged. Orexin-IR projections to the rostral VTA were decreased in all treatment groups compared to controls and there was an overall interaction between maternal and post-weaning HFD exposure. Interestingly, maternal HFD exposure, in isolation and when combined with postnatal HFD consumption, also appeared to decrease orexin-IR fiber output to the rostral VTA indicating this alteration may be programmed developmentally. Despite changes in orexin output and the number of detectable TH-IR cells, there was no effect of maternal or post-weaning HFD consumption on the percentage of TH-IR receiving close appositions from orexin-IR fibers. Therefore, it remains unclear whether this decrease in orexin projections specifically alters dopamine signaling. These findings indicate that communication between the LH and VTA may be altered with early exposure to a HFD and could underlie the increased hedonic feeding characterized in this model (19).

Although the number of TH-IR cells are increased in the VTA with post-weaning HFD exposure, it is unclear what this means for the release of dopamine from these neurons. Previous work from our laboratory has observed that maternal HFD exposure decreased the amount of TH-IR fibers in the prefrontal cortex (19), which is a critical VTA projection for the regulation of higher order reward signaling and hedonic appetite regulation (30, 31). In addition, dopamine receptors implicated in feeding (D1 and D2) were also downregulated in the prefrontal cortex, indicating the net effect of maternal HFD exposure was to decrease dopamine signaling in the prefrontal cortex which may reduce the “reward” value for a given stimulus. Maternal HFD decreased orexin fiber output to the VTA in the current study; therefore, it is possible that decreases in orexin release in the VTA trigger the decreased dopamine signaling occurring in the prefrontal cortex. Both rodent and primate offspring from HFD-consuming dams display increased preference for foods high in sugar and fat (19, 32), indicating that they may need to consume more of these foods to get the same reward sensation. We hypothesized that diminished dopamine signal may be one mechanism that drives increased hedonic intake, since more stimulus may be required to reach the same neurological reward signal. Interestingly, this impact of HFD exposure on orexin projections to the VTA did not seem to be specific to early maternal exposure, since animals receiving the HFD only during the post-weaning period also showed this change. The effects of post-weaning HFD consumption were not investigated in the previous study; therefore, the impact of post-weaning HFD on TH-IR fiber output to the prefrontal cortex and whether this also increases the drive for hedonic intake is currently unknown.

Orexin-IR cell number was increased with post-weaning HFD consumption, similar to previous reports in rodents (33). However, this report in rodents also found increases in orexin mRNA expression, which were not observed in the current study. It is possible that quantification of mRNA area in the current study missed changes in intensity within individual cells; however, intensity is difficult to accurately quantify due to the frequent occurrence of saturation. Despite the increase in orexin cell number, projections to the VTA were decreased. One hypothesis is that the increase in orexin cell numbers represents those cells projecting to other areas, such as the various regions of the hypothalamus and/or other brainstem regions such as the locus coerelus, all of which have previously been shown to receive orexin projections in nonhuman primates (34–36). The current study's finding of decreased immunoreactivity in the VTA with early HFD exposure led to the current hypothesis that orexin signaling to the VTA may be decreased in the current model. With decreased orexin output to the VTA and decreased TH output to the prefrontal cortex described previously (19), this indicates that early exposure to HFD may dampen the reward neurocircuitry at two distinct nuclei. No differences in close appositions between orexin fibers and dopamine neurons was observed in the current study, indicating that other factors may also down-regulate dopamine production and/or release. This conclusion is supported by the finding that the mHFDpCTR group demonstrated decreased orexin fiber density in the VTA but no difference in TH-IR cell numbers. It should be noted that close apposition analysis only examined a subset of cells and subtle differences in orexin-dopamine contacts could be missed. Electrophysiological studies examining endogenous orexin tone on dopamine neurons following early HFD-exposure could better validate the current findings.

Interestingly, although MCH neurons are hypothesized to provide input to VTA dopamine neurons in the rodent (37), this connection was largely absent in the current study, providing to our knowledge the first evidence that this projection is not present in nonhuman primates. This finding is consistent with electrophysiological findings in rodents, demonstrating a lack of MCH effects on VTA neuron firing frequency in both dopaminergic and non-dopaminergic neurons (21). Another neuropeptide of the lateral hypothalamus called neurotensin is also known to send direct projections to VTA dopamine neurons in the rodent, and future studies can explore whether this connection is altered with early HFD exposure. Similarly, there are several other known projections to dopamine neurons beyond the LH, including neuropeptides like oxytocin and vasopressin of the paraventricular hypothalamus and neurotransmitters like serotonin, glutamate and GABA which project from the dorsal raphe (38). Future studies in this model can explore how these additional inputs to dopamine neurons are affected by early HFD exposure and may regulate hedonic feeding in primates.

It should be noted that the current study has examined changes in neurocircuitry driven by maternal HFD consumption and not obesity *per-se*. Like humans, monkeys display different sensitivities to the obesogenic HFD, and not all HFD-fed dams in the current model were obese and not all CTR-fed dams were lean, as previously reported (8). Previous work has found that maternal obesity and maternal HFD consumption may elicit different effects on offspring neuroanatomy and metabolism (8, 19); however, a limited sample size prevented the separation of these effects in the current study. Nevertheless, the current study provides evidence that both maternal and post-weaning HFD consumption may alter neurocircuits regulating reward and increase hedonic feeding in offspring. This change does not occur in isolation and it is likely that multiple organ systems contribute to the increased obesity risk in offspring from obese mothers. As more animals from this model become available we hope to correlate expression changes in the brain to physiological outcomes like hedonic food intake to determine which changes may have the greatest impact on metabolic health. Recent work has found evidence that HFD exposure can alter oocytes in females resulting in mitochondrial dysfunction that is passed on to future generations (39). In addition, maternal obesity has also been shown to alter placental function which may disrupt nutrient exchange between mother and fetus (40). Fetal defects in the liver and muscle, which are critical for glucose homeostasis are also altered with maternal HFD exposure (8). The current nonhuman primate model will continue to be utilized for a collaborative whole organism approach to determine how maternal HFD-induced changes across multiple tissues are interconnected and their relative contribution toward obesity risk in offspring.

## Author contributions

CT wrote the manuscript, contributed to data analysis and interpretation. AA, SL, and MK performed data collection and analysis. ES contributed to study design. PK contributed to study design, data interpretation, and critical discussion of the manuscript.

### Conflict of interest statement

The authors declare that the research was conducted in the absence of any commercial or financial relationships that could be construed as a potential conflict of interest.
