# Supplementary material for: Early High-Fat Diet Exposure Causes Dysregulation of the Orexin and Dopamine Neuronal Populations in Nonhuman Primates
Source: Front Endocrinol (Lausanne). 2018 Sep 10;9:508. doi: 10.3389/fendo.2018.00508 (PMC6143816; doi:10.3389/fendo.2018.00508)
Supplement: Supplementary file 1 [file Table_1.DOCX]

| DAMS | | | | |
| --- | --- | --- | --- | --- |
|  | *mCTR (n)* | | *mHFD (n)* | |
| Weight (kg) | 8.6 ± 0.4 (*17*) | | 11.8 ± 0.7 (*16*) * | |
| Age (yrs) | 7.1 ± 0.5 (*17*) | | 8.8 ± 0.3 (*16*)* | |
| Percent Body Fat | 16 ± 2.4 (*11*) | | 31 ± 4.4 (*7*)* | |
| ivGTT Glucose AUC | 9101 ± 488 (*11)* | | 7494 ± 602 (*16*) | |
| ivGTT Insulin AUC | 3313 ± 551 (*11)* | | 8959 ± 1790 (*16*)* | |
| OFFSPRING | | | | |
|  | *mCTRpCTR (n)* | *mCTRpHFD (n)* | *mHFDpCTR (n)* | *mHFDpHFD (n)* |
| Age at weaning (days) | 261 ± 5 *(8)* | 263 ± 9 (*9)* | 246 ± 14 *(8)* | 267 ± 13 *(8)* |
| Weight (kg) | 2.6 ± 0.1 *(8)* | 2.7 ± 0.1 (*9)* | 2.6 ± 0.1 *(8)* | 2.8 ± 0.2 *(8)* |
| ivGTT Glucose AUC | 8743 ± 267 *(7)* | 8406 ± 557 (*9)* | 8728 ± 514 *(8)* | 7997 ± 446 *(8)* |
| ivGTT Insulin AUC | 1538 ± 222 *(8)* | 1761 ± 288 *(8)* | 1705 ± 288 *(8)* | 1774 ± 220 *(8)* |

Supplemental Table 1. Basic characteristics of dams and offspring utilized for the current study

Data presented as mean ± SEM

ivGTT – intravenous glucose tolerance test

AUC – area under the curve, calculated from zero

* student’s t-test between mCTR and mHFD p<0.05
